# Humoral Immune Responses to a Single Allele PfAMA1 Vaccine in Healthy Malaria-Naïve Adults

**DOI:** 10.1371/journal.pone.0038898

**Published:** 2012-06-29

**Authors:** Edmond J. Remarque, Meta Roestenberg, Sumera Younis, Vanessa Walraven, Nicole van der Werff, Bart W. Faber, Odile Leroy, Robert Sauerwein, Clemens H. M. Kocken, Alan W. Thomas

**Affiliations:** 1 Biomedical Primate Research Centre, Rijswijk, The Netherlands; 2 Radboud University Nijmegen Medical Centre, Nijmegen, The Netherlands; 3 European Vaccine Initiative, Heidelberg, Germany; Burnet Institute, Australia

## Abstract

**Plasmodium falciparum:**

apical membrane antigen 1 (AMA1) is a candidate malaria vaccine antigen expressed on merozoites and sporozoites. The polymorphic nature of AMA1 may compromise vaccine induced protection. The humoral response induced by two dosages (10 and 50 µg) of a single allele AMA1 antigen (FVO) formulated with Alhydrogel, Montanide ISA 720 or AS02 was investigated in 47 malaria-naïve adult volunteers. Volunteers were vaccinated 3 times at 4 weekly intervals and serum samples obtained four weeks after the third immunization were analysed for (i) Antibody responses to various allelic variants, (ii) Domain specificity, (iii) Avidity, (iv) IgG subclass levels, by ELISA and (v) functionality of antibody responses by Growth Inhibition Assay (GIA). About half of the antibodies induced by vaccination cross reacted with heterologous AMA1 alleles. The choice of adjuvant determined the magnitude of the antibody response, but had only a marginal influence on specificity, avidity, domain recognition or subclass responses. The highest antibody responses were observed for AMA1 formulated with AS02. The Growth Inhibition Assay activity of the antibodies was proportional to the amount of antigen specific IgG and the functional capacity of the antibodies was similar for heterologous AMA1-expressing laboratory strains.

**Trial Registration:**

ClinicalTrials.gov NCT00730782

## Introduction

Malaria is a serious public health problem causing high levels of morbidity and mortality in malaria-endemic regions [1]. An effective malaria vaccine will contribute to reducing the burden of malaria in addition to existing measures like insecticide-treated bed nets, antimalarials and vector control.

Apical Membrane Antigen 1 (AMA1) is a promising malaria vaccine candidate (reviewed in [2]), expressed on merozoites and sporozoites as a type I integral membrane protein [3], [4] and is initially located in the micronemes [3], [5]. In *Plasmodium falciparum* AMA1 is expressed as an 83 kDa protein and relocates as a 66 kDa protein to the parasite surface following cleavage of the pro-sequence at the time of invasion [6], where it is subsequently shed as 44 and 48 kDa forms [6]. The AMA1 ectodomain consists of an N-terminal pro-sequence and three tightly interacting domains [7], [8]. AMA1 is believed to play an essential role in red blood cell invasion [9] and may also be implicated in liver cell invasion by sporozoites [4]. The protective efficacy of AMA1-based vaccines has been demonstrated in numerous mouse and simian models (reviewed in [2]).

AMA1 is a polymorphic antigen [10], and this polymorphism is entirely due to single amino acid substitutions [7]. These polymorphisms have been found to be restricted to the surface of AMA1, predominantly mapping to one molecular face [7], [8], [11]. Studies with the rodent malaria *P. chabaudi* have shown that polymorphism in AMA1 can ablate vaccine efficacy [12]. Rabbit immunisation studies have demonstrated that, although antibodies raised against one PfAMA1 allele show excellent inhibition of strains expressing a homologous allele, strains expressing a heterologous PfAMA1 allele are inhibited to a variable lesser degree depending on the antigenic differences and their locations [13]–[17], suggesting that PfAMA1 polymorphism reduces the efficacy of PfAMA1 based vaccines. Results from a Phase IIb vaccine study in Malian children suggests that the specificity of the AMA1 immune response is crucial in protection [18].

Most of the data addressing the impact of polymorphism have been generated in laboratory animals and relatively little is known from human vaccination studies. A phase Ia study with 10 or 50 µg of a single allele vaccine (*Pf*AMA1 FVO-strain, amino acids 25–545) formulated with three adjuvants [19] in malaria-naïve subjects offers a unique opportunity to perform an in depth analysis of antibody responses in humans. Total IgG and GIA responses to the homologous antigen have been reported previously [19]. The analysis reported here comprises ELISA and Growth Inhibition Assay (GIA) titres to the homologous and heterologous AMA1 antigens, domain specificity, subclass distribution, avidity and the relation between GIA and ELISA titres after 3 immunisations with *Pf*AMA1 FVO 25–545. In addition, it also offers the opportunity to investigate the effect of various adjuvants on the quality and quantity of the humoral immune response.

## Materials and Methods

### Participants

Fifty-six malaria-naïve healthy male study participants were recruited at the Radboud University Nijmegen Medical Centre as previously reported [19]. The Dutch regulatory authorities advised to only enrol males, because no reproductive toxicology data were available for the vaccine formulations. A total of 47 subjects completing all 3 immunisations were included for the per protocol analysis in the current paper [19]. A total of 8 subjects were withdrawn because of adverse events (1 Rash, 7 Erythema). Of the 7 erythema cases 2 and 4 occurred in the AS02 10 and 50 µg groups, respectively [19]. One volunteer was withdrawn because of concomitant hepatitis vaccination [19]. The characteristics of the subjects at the time of first vaccination are presented in Table 1. All volunteers provided written informed consent. The study was approved by the Institutional Review Board (CMO Regio Arnhem-Nijmegen, 2005/015). The study was conducted in accordance with the Declaration of Helsinki principles for the conduct of clinical trials and the International Committee of Harmonization Good Clinical Practice Guidelines and registered at www.clinicaltrials.gov (NCT00730782).

**Table 1:** Characteristics of the subjects at the time of first vaccination (per protocol). Age is presented as mean ± standard deviation.

Adjuvant	Dose	N	Age	Minimum	Maximum
Alhydrogel	10	9	23.3±2.9	21.1	29.5
Alhydrogel	50	10	23.2±2.1	19.4	26.9
ISA720	10	8	23.1±5.2	18.5	33.7
ISA720	50	9	23.2±3.6	19.7	31.1
AS02	10	7	24.5±8.3	20.0	42.8
AS02	50	4	24.3±4.8	20.5	31.3

### Vaccines, Vaccination and Blood Samples

Clinical grade *Pf*AMA1 FVO[25–545] consisting of *P. falciparum* FVO-strain AMA1 ectodomain (amino acids 25 to 545) was produced as previously described [20]. The cGMP (current Good Manufacturing Practice) produced *Pf*AMA1 FVO[25–545] was available in multidose vials containing 120 µg lyophilised AMA1 (44 µg EDTA, 180 µg saccharose and 120 µg NaHCO_3_, Lot B) or 62.5 µg lyophilised AMA1 (23 µg EDTA, 25 mg saccharose, 226 µg K_2_HPO_4_ and 187 µg NaH_2_PO_4_, Lot C). *Pf*AMA1 vaccines of 0.5 mL were prepared at two dosages (10 and 50 µg) with three different adjuvants (Alhydrogel, Montanide ISA 720 or AS02) as previously described [19]. Alhydrogel and Montanide ISA 720 vaccines were prepared with lot B *Pf*AMA1 and AS02 vaccines were prepared with lot C *Pf*AMA1; the excipientation of lot C was specifically optimised for compatibility with AS02. Formulated vaccines were kept at 4°C for a maximum of six hours until administration. Vaccine formulations were tested for stability as previously described [20] and all fulfilled the pre-set specifications. Immunisations were given intramuscularly in the deltoid region of alternate arms at days 0, 28 and 56. Blood was collected in Vacutainer™ CPT tubes (Becton and Dickinson), plasma collected after centrifugation and stored at −20°C.

### Quantification ELISA for Total IgG and IgG Subclasses

**Figure 1:**
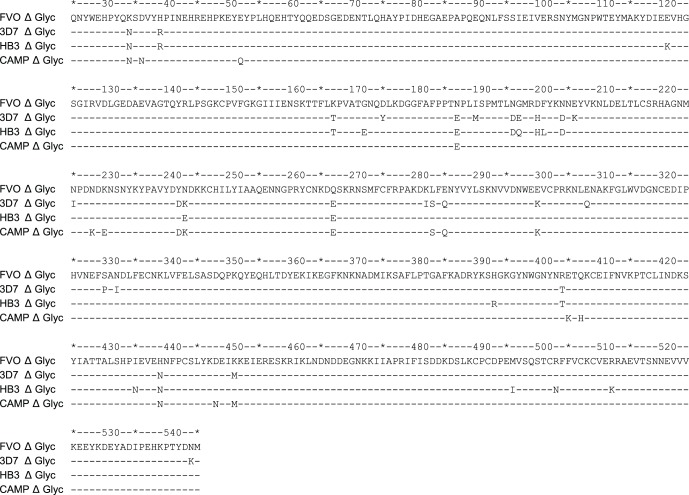
Alignment of AMA1 protein sequences amino acids 25 through 545 used for ELISA measurements. Amino acid positions are indicated above the sequences for each decimal and every fifth position is indicated by an asterisk (*). Each line represents an AMA1 allele, for FVO, 3D7, HB3 and CAMP, respectively. Δ glyc indicates that it concerns sequences that have been mutagenised to avoid potential N-glycosylation sites. Potential N-glycosylation sited were changed (six for FVO, 3D7 and CAMP and five for HB3) to avoid unwanted N-glycosylation: N 162 K (For FVO, 3D7 and CAMP. HB3 AMA1 has K at position 162). T 288 V, S 273 D, N 422 D, S 423 K and N 499 Q.

Enzyme-linked immunosorbent assay (ELISA) was performed in duplicate on plasma samples in 96 well flat-bottomed microtitre plates (Greiner, Alphen a/d Rijn, The Netherlands), coated with 1 µg/mL purified AMA1 antigens according to published methods [10]. The sequences of the four *Pichia pastoris-* expressed AMA1 proteins used for ELISA are shown in Figure 1, potential N-glycosylation sites were removed as previously described [10], [15] and proteins were produced as previously described [20]. The *P. pastoris*-expressed AMA1 from 3D7, HB3 and CAMP used in the ELISA’s differ by 26 (2, 17, 5 and 2 for prodomain and domains I, II and III, respectively), 20 (2, 11, 4, 3 for prodomain and domains I, II and III, respectively) and 17 (3, 9, 3, 2 for prodomain and domains I, II and III, respectively) amino acid positions in the ectodomains (aa 25–545) (Figure 1). The secondary antibody for total IgG was goat anti-human IgG conjugated to alkaline phosphatase (Pierce, Rockford, IL). The reagents used for the total IgG ELISA to the AMA1 alleles were the same except for the coating antigens and the ELISA’s were all performed in one run and the coating antigens were of comparable quality and purity. As all ELISA’s were done in one run with comparable reagents it is assumed that an OD of 1 over blank represents equal amounts of antibodies. For the IgG subclasses the mouse monoclonals conjugated to horseradish peroxidase were used: M1328 (clone MH161-1), M1329 (Clone MH162-1), M1330 (Clone MH163-1) and M1331 (Clone MH164-1) for IgG1, IgG2, IgG3 and IgG4, respectively (Sanquin, Amsterdam, The Netherlands). These monoclonal antibodies are confirmed to be specific for the designated subclass and show no cross reactivity with other IgG subclasses at the recommended working dilutions. A serum pool from >50 exposed African adults was included on each plate and antibody levels in the unknowns were calculated using a four-parameter fit. Titres are expressed as arbitrary units, where 1 AU yields an OD of 1 over background. Thus the amount of AU of a sample is the reciprocal dilution at which an OD of 1 over background will be achieved. One arbitrary antibody unit for total IgG corresponds to approximately 15 ng mL^−1^ of antigen specific IgG.

### Competition ELISA

Competition ELISA was performed as previously described [16]. Dilutions that resulted in 2 AU (yielding an OD of about 1.5 over blank) were calculated for each plasma sample and used in the subsequent antigen competition assay. The assay involved co-incubation of different allelic forms of PfAMA1, or PfAMA1-FVO domains with test plasma in plates coated with PfAMA1 FVO allele, such that competition occurs between the added (competitor) antigens and the coated antigen for binding to test IgG’s. For the AMA1 allelic forms the competitor/soluble antigens were titrated 3-fold over 7 wells from 100 to 0.137 µg mL^−1^ with PfAMA1 from the FVO, 3D7, HB3 or CAMP alleles (Figure 1). For the PfAMA1-FVO domains competition ELISA, plates were coated with FVO AMA1 amino acids 97 through 545 (Domains I, II and III) a fixed concentration of 30 µg mL^−1^ of various FVO AMA1 domain constructs were used as competitors (pro-DI-II-III aa 25–545, DI-II aa 97–442, DII-III aa 303–545 and DII aa 303–442). The appropriately diluted plasma was then added and after incubation for 2 h, plates were developed as described above. Values are presented as the fraction remaining relative to the initial amount.

### Avidity ELISA

The avidities of the antibodies were determined by sodium isothiocyanate (NaSCN) elution ELISA as previously described [16]. Briefly, microtitre plates were coated with AMA1 variant proteins (Figure 1) as described above, and after blocking, incubated with a pre-determined titre (1 AU) of sera for 1 h. Plates were then washed and incubated with an increasing concentration of NaSCN (ranging from 0 to 3.0 M in 0.25 M steps) in duplicate wells for 15 min. Plates were washed and developed with goat anti-human IgG alkaline phosphatase conjugate and substrate as previously described [16]. The avidity index is expressed as the concentration of NaSCN required for 50% dissociation of bound antibodies (relative to duplicate wells without NaSCN).

### Growth Inhibition Assay (GIA)

Antibodies used for growth inhibition assays (GIA) were purified from CPT plasma on protein A columns (Immunopure Plus Pierce, St Louis, MO, USA), exchanged into RPMI 1640 using Amicon Ultra-15 concentrators (30 kDa cutoff, Millipore, Ireland), filter-sterilised and stored at −20°C until use. IgG concentrations were determined using a Nanodrop ND-1000 spectrophotometer (Nanodrop Technologies, Wilmington, DE, USA). *P. falciparum* strains FCR3, 3D7 and HB3 were cultured *in vitro* using standard culture techniques in an atmosphere of 5% CO_2_, 5% O_2_ and 90% N_2_. FCR3 AMA1 (accession no. M34553) differs by one amino acid in the pro-sequence (D36G) from FVO AMA1 (accession no. AJ277646) and is considered the homologous AMA1 antigen for the FVO allele. The ectodomain (amino acids 25–545) of 3D7 (accession no. U65407) differs by 26 amino acids (2, 17, 5 and 2 for prodomain and domains I, II and III, respectively) from FVO and the ectodomain of HB3 (accession no. U33277) differs by 21 amino acids (2, 12, 4 and 3 for prodomain and domains I, II and III, respectively) from FVO.

The GIA was performed as previously described [14]. Briefly, the effect of purified IgG antibodies on *in vitro* parasite growth was evaluated at two IgG concentrations (5 and 10 mg/mL, respectively) and each participants pre-immune IgG was used as negative control. A IgG concentration of 10 mg mL^−1^ approximates the amount of IgG (9.5 to 11.5 mg mL^−1^) found in undiluted human plasma [21]. Samples were run in triplicate using 96 well flat-bottomed plates with alanine-synchronized cultures of *P. falciparum* schizonts at an initial parasitaemia of 0.2–0.4%, a haematocrit of 2.0% and a final volume of 100 µL. After 40 to 42 hours, cultures were resuspended, and 50 µL was transferred into 200 µL ice-cold PBS. The cultures were then centrifuged, the supernatant removed and the plates were frozen. Parasite growth was assessed by measuring parasite lactate dehydrogenase levels with the lactate diaphorase APAD substrate system, and plates were read at 655 nm after 30 min incubation in the dark. Parasite growth inhibition was expressed as: 100 × (1– (OD_655_ Day84– OD_655_ RBC)/(OD_655_ Day0– OD_655_ RBC)), Where: OD_655_Day84 is the OD_655_ for IgG purified from day 84 plasma, OD_655_Day0 is the OD_655_ for IgG purified from day 0 plasma and OD_655_RBC is the average OD_655_ of RBC control wells. The data are presented as the arithmetic mean % inhibition from each sample triplicate.

**Figure 2:**
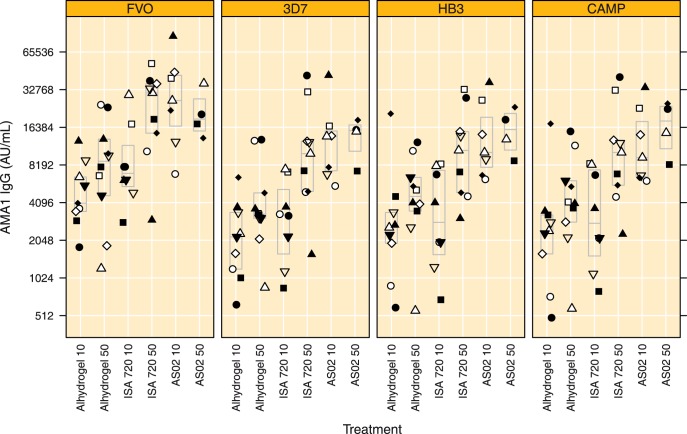
IgG levels to the vaccine allele and three variant AMA1 alleles in plasma obtained on day 84. IgG antibody levels to the vaccine antigen (FVO) and 3 heterologous AMA1 alleles (3D7, HB3 and CAMP). Values for each subject within a treatment group are indicated by a specific symbol (same symbol within each treatment group is same subject throughout graphs 2, 4, 5, 6 and 7), boxes indicate median and quartile ranges. Treatment groups are indicated by Adjuvant name and AMA1 dose, respectively.

### Statistics

Antibody titres (IgG and IgG subclasses) were log-transformed to obtain normality and are presented as geometric means with 95% confidence intervals. GIA titres, IgG avidity and levels of depletion were approximately normally distributed and are therefore presented as arithmetic means with 95% confidence intervals. The statistical significance of between group differences was initially evaluated using one way (comparing the 6 adjuvant-dose groups directly) Analysis of Variance (ANOVA). Significant between group differences were further evaluated by Tukey’s Honest Significant Difference post-hoc test which applies a correction on the p-value for multiple comparisons and provides estimates with 95% confidence intervals (95% CI) for the between group comparisons.

The results from the competition ELISA were analysed using non-linear mixed effects models (NLME). Where the fraction of antibody remaining bound at increasing competitor concentration was modeled separately for each subject by the following formula describing a sigmoid: Fraction remaining  = 1/[1+ Exp((Ln IC_50_– Ln IgG ) * Slope) ]. As Slope is difficult to interpret, it is expressed as the fold-increase in soluble antigen concentration required to change the fraction remaining bound from 90 to 50% or from 50 to 10%. This fold-increase can be calculated by the following formula e^(2.197/Slope)^.

NLME models with and with and without treatment effects were assessed and the model with the lowest Aikake Information Criterion (AIC) were accepted as best fitting model. The IC_50_ and fold increase in competitor required to change the fraction bound from 90 to 50% were calculated from the regression results.

The relation between GIA and IgG titres was investigated by non linear least squares regression using the following formula: GIA  = 100 * 1/[1+ Exp((Ln IC_50_– Ln IgG ) * Slope) ], where IgG is the antibody titre, IC_50_ represents the IgG concentration yielding 50% inhibition and slope is a parameter indicating the steepness of the curve, with steeper curves at higher (absolute) values. Comparisons of IC_50_ and slope values for the various GIA strains were done by incorporating dummy variables for both parameters for both strains in the non linear least squares regression model.

**Figure 3:**
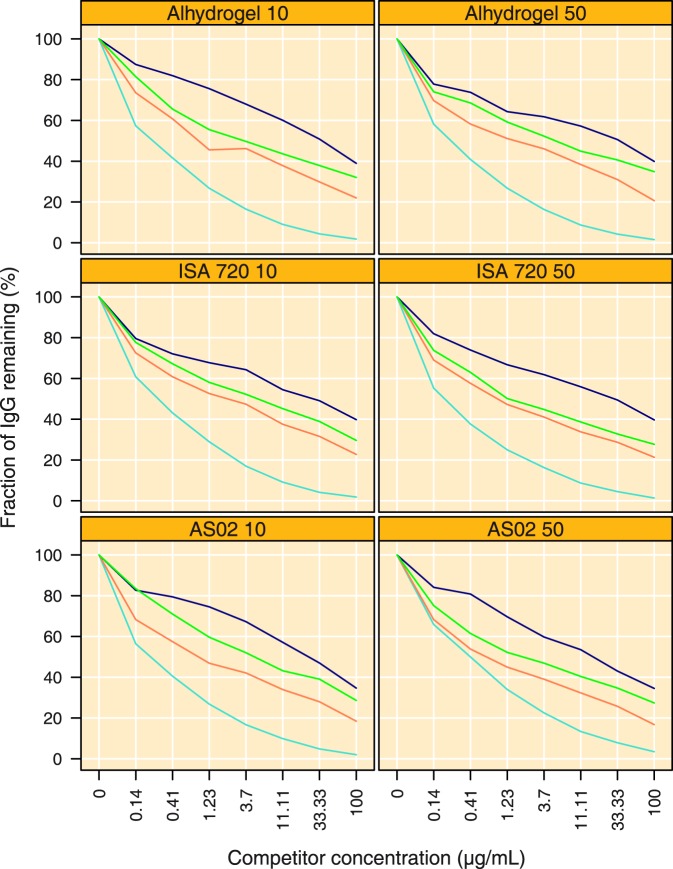
Competition ELISA using four different competitor antigens on FVO coated plates in plasma obtained on day 84. Concentration of competitor antigen is depicted on the x-axis (µg mL^−1^) and the fraction remaining bound (mean from all individuals in a treatment group) to the ELISA plate is depicted on the y-axis (%). Light Blue  =  FVO AMA1 (homologous), Orange  =  HB3 AMA1, Green  =  CAMP AMA1 and Dark Blue  = 3D7 AMA1. Treatment groups are indicated by Adjuvant name and AMA1 dose, respectively.

## Results

### IgG Responses to AMA1 Alleles

Before vaccination all 47 available plasma samples were negative for AMA1 IgG antibodies (data not shown) [19]. All available plasma samples obtained 4 weeks after the third vaccination (Day 84) were titrated in a single laboratory run on 4 different AMA1 variants; IgG antibody titres are depicted in Figure 2. The results obtained for the IgG titres against FVO AMA1 in the 47 day 84 samples are in agreement with those previously reported (Spearman’s Rho = 0.943, p<0.0001) [19]. IgG antibody levels were lowest in the Alhydrogel groups (10 and 50 µg) and in the Montanide ISA 720 10 µg group as compared to the Montanide ISA 720 50 µg and both AS02 groups, this trend was consistently observed for all AMA1 variants under investigation (Figure 2). The IgG antibody levels to the heterologous AMA1 (3D7, HB3 and CAMP) variants, were approximately 50% lower as compared to the homologous FVO AMA1 (Figure 2). IgG antibody levels to all AMA1 variants under investigation were 4 to 5 fold higher in the Montanide ISA 720 50 µg and AS02 10 µg groups as compared to the Alhydrogel 10 µg group (Figure 2, all p<0.02).

**Figure 4:**
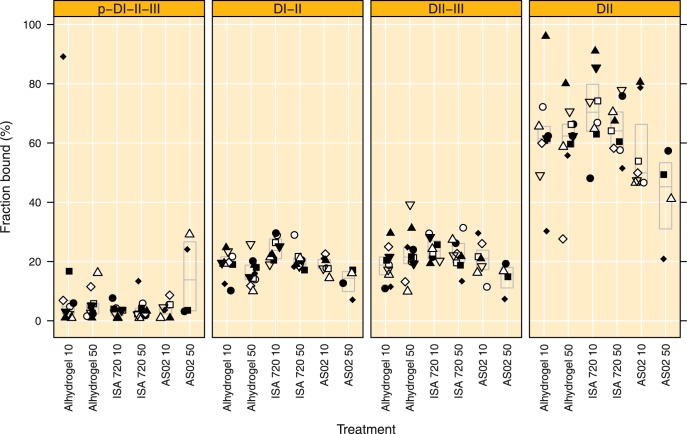
Competition ELISA using AMA1 domain constructs in plasma obtained on day 84. The fraction of antibodies remaining bound after competition with 30 µg of the indicated competitor are depicted on the y-axis, boxes indicate median and quartile ranges. P-DI-II-III  =  FVO AMA1 ectodomain amino acids 25–545, DI-II  =  FVO-AMA1 amino acids 97–442, DII-III  =  FVO AMA1 amino acids 303–545 and DII  =  FVO AMA1 amino acids 303–442. Treatment groups are indicated by Adjuvant name and AMA1 dose, respectively.

The specificity of the IgG responses in day 84 samples was further characterised using competition ELISA, where increasing amounts of competitor antigen were co-incubated with a fixed amount of antibodies. Figure 3 shows that the addition of increasing amounts of antigen reduces the amount of bound antibody for all competitor antigens, with the most pronounced reduction observed for the homologous antigen; the heterologous antigens also reduced the amount of IgG remaining bound, but less efficiently as compared to the homologous antigen. The depletion curves per competitor antigen were similar, as judged by IC_50_ and slope parameters, for the adjuvant and dose groups, suggesting neither adjuvant nor dose markedly influenced antibody specificities (Figure 3). The amount of competitor antigen required to remove 50% of antibodies (IC_50_) was lowest for FVO at 0.23 µg mL^−1^ (95% CI 0.21 to 0.26), 1.47 µg mL^−1^ (95% CI 1.08 to 2.02) for HB3, 4.39 µg mL^−1^ (95% CI 2.93 to 6.57) for CAMP and 20.15 µg mL^−1^ (95% CI 14.17 to 28.65) for 3D7 (Figure 3). The increase in competitor antigen concentration required to reduce the remaining amount of bound antibodies from 50 to 10% was 38-fold (95% CI 34 to 43) for FVO AMA1. The slopes for the heterologous AMA1 proteins indicated that a much larger increase in competitor concentration was required to reduce the amount of bound antibodies from 50 to 10%: 1015-fold (95% CI 702 to 1535), 1328-fold (95% CI 920 to 2001) and 910-fold (95% CI 471 to 2058) for HB3, CAMP and 3D7 AMA1, respectively (Figure 3).

**Figure 5:**
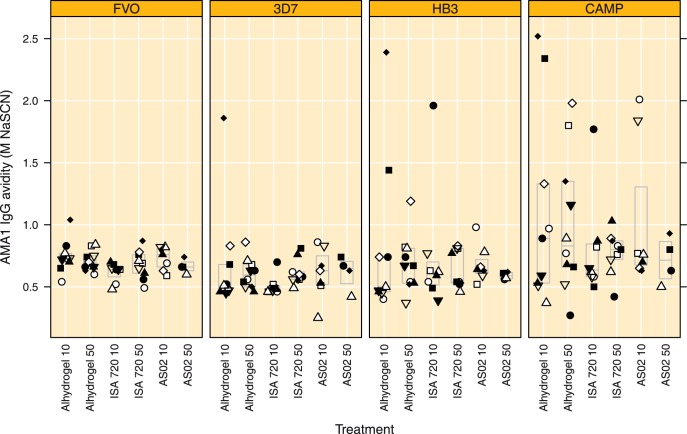
Antibody avidity to the vaccine allele and three variant alleles in plasma obtained on day 84, boxes indicate median and quartile ranges. Antibody avidity is expressed as the amount of NaSCN required to dissociate 50% of bound antibodies. Treatment groups are indicated by Adjuvant name and AMA1 dose, respectively.

### IgG Responses to AMA1 Domains

The domain specificity of the antibody response at day 84 was investigated using competition ELISA’s, where fixed amounts (30 µg) of PfAMA1-FVO domains were added. Figure 4 shows that 7.2% (95% CI 3.3 to 11.2) of the IgG remained bound after the addition of 30 µg of the full length antigen (p-DI-II-III), indicating over 90% depletion and suggesting that the 30 µg mL^−1^ is close to saturation. The fraction of IgG remaining after the addition of the full length antigen did not differ for the groups (p = 0.36). After addition of domains I-II approximately 19.0% (95% CI 17.6 to 20.5) of the IgG remained bound, indicating more than 80% depletion. The addition of domains I-II resulted in significant between group differences in the fraction remaining bound (p = 0.0009, ANOVA). More IgG remained bound In the Montanide ISA 720 10 µg group when compared to the Alhydrogel 50 µg and AS02 50 µg groups, (7.8% (95% CI 2.0 to 13.6, p  = 0.0030, Tukey HSD) and 10.9 (95% CI 3.4 to 18.4. p = 0.0011, Tukey HSD). A similar trend, albeit not significant, was observed when comparing the Montanide ISA 720 50 µg group with the AS02 50 µg group (7.2% difference, 95% CI −0.17 to 14.5, p = 0.059, Tukey HSD). This suggests that Montanide ISA 720 induces more antibodies directed against domain III. No significant differences were observed between the treatment groups following depletion with domains II-III or domain II only (p values 0.16 and 0.051, respectively, ANOVA). The average amount of IgG remaining bound was 21.1% (95% CI 19.2 to 23.0) and 61.5% (95% CI 57.1 to 66.0) for domains II-III and domain II, respectively.

### Antibody Avidity

Antibody avidity in individual plasma samples collected on day 84 was quantified using a sodium thiocyanate (NaSCN) elution ELISA and expressed as the concentration of NaSCN required to reduce the amount of bound antibody by 50%, shown in Figure 5. Adjuvant or antigen dose did not significantly influence the average avidity to the AMA1 variants under investigation (p = 0.169, p = 0.725, p = 0.864 and p = 0.609 for FVO, 3D7, HB3 and CAMP, respectively). The mean concentration of thiocyanate required for 50% elution was 0.694 M (95% CI 0.660 to 0.725) for the homologous FVO AMA1. For the heterologous antigens these were: 0.609 M (95% CI 0.542 to 0.676), 0.711 M (95% CI 0.602 to 0.820) and 0.929 M (95% CI 0.775 to 1.083) for 3D7, HB3 and CAMP AMA1, respectively. Antibody avidity to the 3D7 AMA1 was significantly lower than to the homologous FVO AMA1 (p = 0.006, paired t-test). By contrast, antibody avidity to the CAMP AMA1 was significantly higher than to the homologous FVO AMA1 (p = 0.002, paired t-test). Antibody avidities to the HB3 allele were similar to those observed for the FVO allele (p = 0.742, paired t-test).

**Figure 6:**
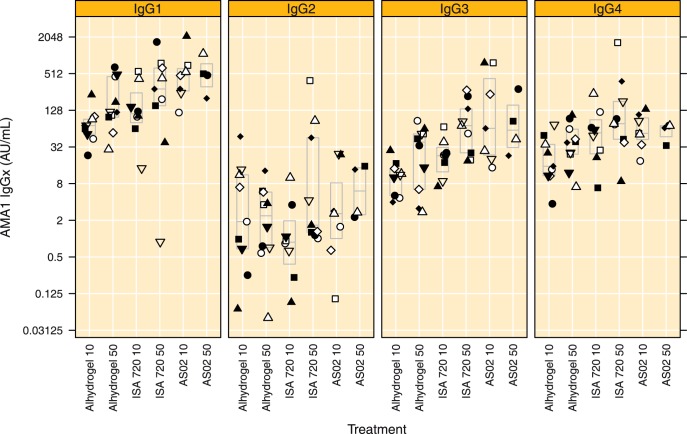
IgG subclasses to the vaccine antigen in plasma obtained on day 84, boxes indicate median and quartile ranges. Treatment groups are indicated by Adjuvant name and AMA1 dose, respectively.

### Subclass Distribution

IgG subclass levels to the homologous FVO AMA1 allele determined in plasma samples taken on days 0 and 84. Pre-vaccination subclass levels were low and no significant differences were observed between the treatment groups. Following vaccination IgG1, IgG2, IgG3 and IgG4 levels all increased significantly (p<0.001, Wilcoxon signed rank test). Day 84 subclass levels are shown in Figure 6. The ranking of IgG subclasses in descending titre order was IgG1> IgG3 ≈ IgG4> IgG2. IgG1 and IgG3 antibody levels showed a similar pattern as to what was observed for total IgG, with lowest titers in the Alhydrogel groups (10 and 50 µg) and in the Montanide ISA 720 10 µg group as compared to the Montanide ISA 720 50 µg and both AS02 groups (Figure 6). Interestingly, titre differences between the treatment arms were slightly higher for IgG3 as compared to IgG1 (Figure 6).

**Figure 7:**
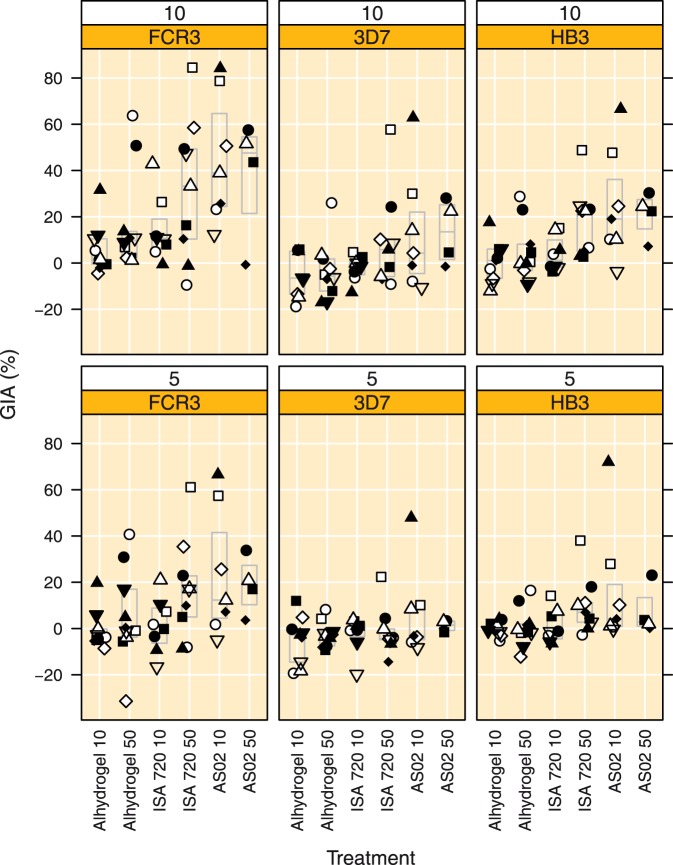
GIA to 3 laboratory strains (FCR3, homologous and 3D7 and HB3, heterologous) at 10 and 5 mg mL^−1^ total IgG obtained at day 84. Top row: GIA at 10 mg mL^−1^ and bottom row GIA at 5 mg mL^−1^ IgG. Boxes indicate median and quartile ranges. Treatment groups are indicated by Adjuvant name and AMA1 dose, respectively.

### GIA

The results of the Growth Inhibition Assay (GIA) for three laboratory strains obtained with IgG fractions purified from plasma samples collected at day 84 are shown in Figure 7. GIA titres determined on the FCR3 laboratory strain expressing an AMA1 similar to the vaccine antigen were below 20% in most (22/27) subjects in the Alhydrogel (10 and 50 µg) and Montanide ISA 720 10 µg groups; only 5 out of 27 subjects had GIA titres higher than 20% at 10 mg mL^−1^ total IgG. By contrast, 14 out of 20 subjects in the Montanide ISA 720 (50 µg) and AS02 (10 and 50 µg) groups had GIA titres exceeding 20% at 10 mg mL^−1^ total IgG (Figure 7). Average GIA responses were significantly higher (38.7%-points, 95% CI 4.7 to 72.6%, p = 0.018, Tukey HSD) in the AS02 10 µg group as compared to the Alhydrogel 10 µg group. The GIA values at 10 mg mL^−1^ total IgG in Montanide ISA 720 and AS02 50 µg groups were also higher than the Alhydrogel 10 µg group, but this failed to achieve statistical significance (p = 0.17 and 0.20, respectively, Tukey HSD, Figure 7). The GIA titres determined on the FCR3 strain at 5 mg mL^−1^ total IgG were lower and showed a ranking similar to what was observed at 10 mg mL^−1^, but none of the between group comparisons reached statistical significance (p = 0.070, ANOVA, Figure 7).

GIA titres determined on the 3D7 laboratory strain at 10 mg mL^−1^ IgG were lower than those to the FCR3 strain at the same total IgG concentration and no significant differences were observed between the treatment groups (p = 0.095, ANOVA). Interestingly, GIA titres on the 3D7 strain at 10 mg mL^−1^ were similar to GIA titres to the homologous FCR3 strain at 5 mg mL^−1^ total IgG (Figure 7). GIA titres on the 3D7 laboratory strain at 5 mg mL^−1^ IgG were low; only 2 out of 47 subjects had GIA titres higher than 20% (Figure 7).

GIA titres determined on the HB3 laboratory strain at 10 mg mL^−1^ IgG were lower than to the FCR3 strain and similar to the titres observed for the 3D7 strain. Significant treatment group differences were observed (p = 0.004, ANOVA). GIA titres in the AS02 10 µg group were significantly higher than in the Alhydrogel 10 µg group (24.6%-points, 95% CI 3.4 to 45.8%, p = 0.014, Tukey HSD) and GIA titres in the AS02 10 µg group tended to be higher as compared to the Alhydrogel 50 µg group (20.5%-points, 95% CI −0.2 to 41.3%, p = 0.053, Tukey HSD) and the Montanide ISA 720 10 µg group (21.2%-points, 95% CI −0.6 to 42.9%, p = 0.061, Tukey HSD). GIA titres on the HB3 laboratory strain at 5 mg mL^−1^ total IgG were low, with only 4 out of 47 subjects having GIA titres of over 20%. GIA titres on the HB3 laboratory strain at 5 mg mL^−1^ IgG in the AS02 10 µg group tended to be higher, similar to what was observed at 10 mg mL^−1^ total IgG, but this failed to reach statistical significance (p = 0.091, ANOVA).

**Figure 8:**
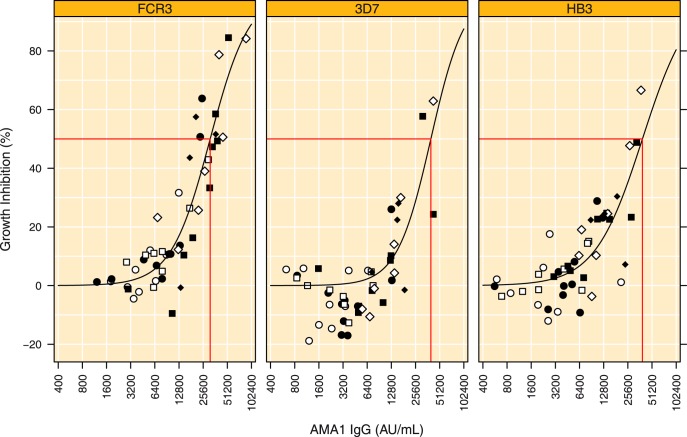
Relation between AMA1-specific IgG titre and GIA titre for the homologous (FCR3) and two heterologous strains (3D7 and HB3). The GIA value for the designated strain measured at 10 mg mL^−1^ total IgG is depicted on the y-axis and the corresponding plasma ELISA titre to the corresponding AMA1 allele is depicted on the x-axis. Estimated IC_50_ values are indicated by red lines. Symbols represent treatment groups: ○  =  Alhydrogel 10 µg, •  =  Alhydrogel 50 µg, □  =  Montanide ISA 720 10 µg, ▪  =  Montanide ISA 720 10 µg, ⋄  =  AS02 10 µg and ⧫  =  AS02 50 µg, where the 10 or 50 µg refers to the AMA1 dose.

### Relation between IgG and GIA Titres

The relation between AMA1 variant-specific IgG and GIA was investigated by modeling sigmoid curves using non-linear least squares regression. The data are graphically presented in Figure 8. The R^2^ values were 0.830 (95% CI 0.714 to 0.902, p<0.0001) for FCR3, 0.632 (95% CI 0.433 to 0.776, p<0.0001) for 3D7 and 0.631 (95% CI 0.431 to 0.775, p<0.0001) for HB3. The IC_50_ values, i.e. the amount of AMA1 specific IgG in plasma required for 50% inhibition were estimated at 31.3 kAU mL^−1^ (95% CI 27.7 to 35.4) for FCR3, 39.8 kAU mL^−1^ (95% CI 30.8 to 51.3) for 3D7 and 38.9 kAU mL^−1^ (95% CI 28.9 to 52.5) for HB3 (Figure 8), which was not significantly different from the value for the FCR3 strain (p = 0.08 and 0.20, respectively). The fold-increase in AMA1 variant specific IgG required to raise the GIA titre from 10 to 50% or from 50 to 90% was 3.5-fold (95% CI 2.7 to 5.0) for FCR3, 2.9-fold (95% CI 2.2 to 5.7) for 3D7 and 4.5-fold (95% CI 3.1 to 9.5) for HB3, the values for the 3D7 and HB3 strains were not significantly different from the value for the FCR3 strain (p = 0.50 and p = 0.33, respectively).

The correlation between antibody avidity and GIA was investigated using (non-parametric) rank correlation tests (Spearman). No significant correlations were observed between antibody avidity to an AMA1 variant and GIA values for a strain expressing a similar AMA1. This may be due to the fact that the variation in avidity values was relatively limited and that the GIA activity is mainly determined by the levels of IgG.

Correlations between IgG subclass levels and GIA to FCR3 were investigated using (non-parametric) rank correlation tests (Spearman’s Rho). Correlations between FCR3 GIA and subclass levels were: rho  = 0.727, p<0.0001 for IgG1, Rho  = 0.027, p = 0.855 for IgG2, Rho  = 0.665, p = <0.0001 for IgG3 and Rho  = 0.480, p = <0.001 for IgG4.

## Discussion

The main finding of this study is that AMA1 antibody responses induced by vaccination of malaria-naïve human volunteers with a single AMA1 variant are biased towards the vaccine allele. This is in agreement with previously published results from a similar human Phase I vaccine trial [22] and observations in rabbits [14], [16]. Approximately half of the IgG induced by vaccination of human volunteers reacts in ELISA with heterologous AMA1 variants, similar to what has been observed in rabbits [14], [16]. The functionality of the antibody response (GIA titre) to heterologous AMA1 expressing strains reflects differences in the quantity of IgG; suggesting that, although absolute antibody levels to heterologous AMA1 variants are lower, these antibodies are, on per mass basis, equally capable of inhibiting parasite growth.

The data presented here also demonstrate that both adjuvant and dose can have a profound impact on antibody levels, while other aspects of the antibody response like avidity, domain recognition, breadth and subclass distribution, are much less influenced by adjuvant or dose. This is, with exception of the subclass distribution, in agreement with what has been observed in rabbit studies [23].

The data presented here show that the widely used adjuvant Alhydrogel yields relatively low antibody levels, and increasing the antigen dose from 10 to 50 µg only marginally improved antibody responses. The data confirm that Alhydrogel is not potent enough to induce high levels of functional antibodies in malaria naïve subjects, as was previously reported [24], [25]. The low potency of Alhydrogel at inducing a functional response is even more pronounced when the functionality of the antibody response is evaluated on strains expressing heterologous AMA1 alleles. Therefore adjuvants more potent than Alhydrogel are definitively required for the induction of functional antibody responses to AMA1 variants not included in a vaccine.

Recently, vaccination induced efficacy against the AMA1 vaccine allele formulated with AS02 in a Phase IIb study was estimated at 64%, whereas overall vaccine efficacy was estimated at 17%, indicating the importance of covering AMA1 variants [18]. Thus, the challenge for an AMA1-based vaccine appears to be with covering AMA1 polymorphism. Theoretically, two options would be available: i) The induction of a broad antibody response and ii) The maximisation of heterologous antibody responses by using potent adjuvants or prime boost strategies combined with a potent adjuvant, such that the response induced with a single variant would be high enough to also be sufficiently functional against heterologous strains. As the latter may not be possible with adjuvants currently available, a more practical approach would be the combination of the induction of the broadest response possible with the highest response possible.

The breadth of the antibody response can be improved by vaccination with a mixture of several AMA1 variants, either naturally occurring [14], [16], [23], [26], or artificial ones [10], [26]. Three artificial diversity covering (DiCo) AMA1 sequences [10] have recently been produced under cGMP and will enter clinical testing in the near future.

The magnitude of the antibody response can be improved by the use of potent adjuvants, as shown here. A novel proprietary adjuvant, CoVaccine HT™, has yielded promising results in rhesus macaques and rabbits [23], [27]. Moreover, *P. knowlesi* AMA1 formulated with CoVaccine HT™ has induced protection against blood-stage challenge with *P. knowlesi* in the rhesus macaque model and the degree of protection was correlated with GIA titre [28]. It would therefore be interesting to combine DiCo AMA1 with CoVaccine HT™ in a Phase I trial.

Antibody avidity was determined with a sodium thiocyanate elution ELISA and average antibody avidities ranged between 0.6 and 0.9 M for the various antigens. These values are lower than what was previously observed in rabbits (0.9 to 1.4 M) [16]. Of note is that the average avidity to the heterologous CAMP AMA1 was higher than the homologous antigen. This could possibly be explained by the fact that half of FVO AMA1-specific antibodies bound to CAMP AMA1 and that this fraction may bind with higher avidity. Conversely, avidities to the heterologous 3D7 AMA1 were lower than the homologous avidities. This may represent antigenic relatedness of the respective AMA1 molecules, with CAMP being more close to FVO AMA1 and 3D7 more distant.

The antibody response to AMA1 appears to be mainly directed against domains I and II, as competition with a construct comprising these domains removes about 80% of antibodies bound. Competition with a domain II-III construct removes a similar amount of antibodies as the I-II construct, suggesting that the majority of the response would be directed against domain II. This is, however, not supported by the depletion obtained by a domain II construct. Of note here is that only constructs including domains I-II induce functionally active antibodies in rabbits [29], [30], suggesting conformational authenticity. The Domain II-III and the domain II constructs both failed to elicit functionally active antibodies in rabbits [29], [30]. The results obtained here warrant the statement that the majority of the antibody response to AMA1 is directed against domains I and II and a further subdivision for these two domains is not possible with the data hitherto obtained. The importance of domains I and II in the antibody response is in agreement with what has been found in rabbits [29], [30].

The subclass distribution found in vaccinated malaria naive volunteers was similar to what was observed in exposed children [31], [32] and reflects the expected subclass distribution for a protein antigen [33]. Antigen dose or adjuvant only marginally influenced the subclass distribution.

In conclusion, vaccination with a single allele AMA1 vaccine induces a humoral immune response that is biased towards the vaccine allele. The magnitude of the response can be enhanced by a potent adjuvant, in contrast other parameters of the humoral response like breadth, avidity and subclass distribution appear much less influenced by the adjuvant. Future vaccine development should focus on improving both breadth and magnitude of antibody responses to AMA1.
